# Expanding clinical roles for nurses to realign the global health workforce with population needs: a commentary

**DOI:** 10.1186/s13584-016-0079-2

**Published:** 2016-06-07

**Authors:** Claudia B. Maier, Linda H. Aiken

**Affiliations:** 2014-15 Harkness & B. Braun Fellow in Healthcare Policy and Practice, Center for Health Outcomes and Policy Research, University of Pennsylvania, School of Nursing, 418 Curie Blvd, Claire M. Fagin Hall, Philadelphia, PA 19104-4217 USA; Department of Healthcare Management, Technische Universität Berlin, Berlin, Germany; Claire M Fagin Professor and Director, Center for Health Outcomes and Policy Research, University of Pennsylvania, 418 Curie Blvd, Claire M. Fagin Hall, 387R, Philadelphia, PA 19104-4217 USA

## Abstract

Many countries, including Israel, face health workforce challenges to meet the needs of their citizens, as chronic conditions increase. Provider shortages and geographical maldistribution are common. Increasing the contribution of nurse practitioners and other advanced practice nursing roles through task-shifting and expansion of scope-of-practice can improve access to care and result in greater workforce efficiency. Israel and many other countries are introducing reforms to expand nurses’ scope-of-practice. Recent international research offers three policy lessons for how countries just beginning to implement reforms could bypass policy barriers to implementation. First, there is substantial evidence on the equivalence in quality of care, patient safety and high consumer acceptance which should move policy debates from if to how to effectively implement new roles in practice. Second, regulatory and finance policies as well as accessible advanced education are essential to facilitate realignment of roles. Third, country experience suggests that advanced practice roles for nurses improve the attractiveness of nursing as a career thus contributing to solving nursing shortages rather than exacerbating them. Designing enabling policy environments and removing barriers will gain in relevance in the future as the demand for high-quality, patient-centered care is increasing.

## Background

Israel, like most other countries in the world, has a health workforce that is struggling to meet the needs of its citizens. Shortages, geographic maldistribution, and specialty misalignment in the context of changing population needs are common [1]. Healthcare work is perceived to be losing its attractiveness as organizational consolidation erodes professional autonomy and relentless cost cutting increases productivity requirements and burnout [2]. Seeking relief, health professionals are on the move globally further detracting from stability of the workforce at home and abroad [3].

The recently published review of global health workforce challenges and the particular challenges in Israel by Aaron & Andrews [4] comes at a crucial moment when the global health workforce has gained political attention worldwide. The World Health Organization has put forward a *Global Strategy on Human Resources for Health: Workforce 2030* for adoption at the May 2016 World Health Assembly [5]. The strategy calls for a decade of action to address the global health workforce deficit, to optimize the composition of the workforce, and respond to the migration of health professionals from rural to urban areas and internationally. Innovative solutions are required to address these challenges. A possible strategy is to expand the roles of non-physician providers, particularly nurse practitioners, with advanced education often at the Master’s degree level, who can effectively provide a large share of primary care services [6].

The evidence on the quality of care of nurse practitioners compared to physicians is substantial, as noted by Aaron & Andrews. In several systematic reviews, nurse-led care substituting -partially or fully- for physicians resulted in at least equivalent quality of care, improved patient satisfaction and reduced hospital admission and mortality in various primary care models compared to physicians [7–9]. Hence, there is a case for introducing advanced practice nursing roles into healthcare systems, if educational standards are met. More research is needed on non-nurse advanced practitioners where there is considerably more variation in education and in role definition.

In response to Israel’s physician shortage, Aaron & Andrews [4] argue that nurse practitioners (NPs) can alleviate workforce shortages if sufficient numbers are educated. Their article draws on international evidence and provides lessons for Israel’s policy and practice developments. The authors provide insights into an emerging international phenomenon which has received limited global attention to date.

### Advanced practice nursing roles globally

Traditional role boundaries between physicians and nurses and other health professionals have been shifting for decades. In many countries, physicians are increasingly specializing and less likely to work in primary care or in rural areas [10]. The increase of chronic conditions has triggered adaptations to service delivery and workforce composition. Traditional occupations like nurses have expanded their scope-of-practice to fill gaps or alleviate shortages.

Advanced roles for nurses performing clinical activities that have traditionally been reserved for physicians are increasing worldwide. We studied the status of task-shifting where nurses work in advanced roles in 39 countries including Europe, Canada, U.S., Australia, and New Zealand [11]. We found that countries with a long tradition and demonstrated experience integrating nurse practitioners or similar roles within their health systems are the U.S., Canada, the four countries of the U.K., the Netherlands, Finland, Ireland, New Zealand and Australia [11]. Several countries in Europe are at early stages of implementation, mirroring the situation in Israel [4]. Israel was not included in our survey but our findings are relevant to informing policy decisions about potential ways forward.

### Implications and policy lessons internationally

We found policy-amenable barriers to role expansion for nurses in virtually all countries. These included a lack of title protection for advanced practice nurses, lack of role clarity, financial barriers in reimbursement, variations in education, unnecessarily restrictive regulations, and resistance by stakeholders, all of which were substantial barriers to implementing new roles in clinical settings [4, 11].

Our investigation showed the important role that governance mechanisms, and in particular, regulation of advanced nursing practice has on implementation. There are large cross-country variations if, how, and when professional titles and scope-of-practice are regulated [11]. Regulation is important as it can facilitate or impede implementation of new roles. Regulation helps formalize the NP role, helps standardize educational requirements, defines practice competence and requirements, and can facilitate payment for NP services, all factors necessary for successful implementation [11, 12]. New adopters of NP roles in the 1960s and 1970s, such as the U.S. and Canada, were understandably concerned about quality of care provided by non-physicians in the absence of much research, and often erred in adopting overly restrictive regulations, which had long-lasting negative consequences including adversely affecting the supply of NPs, hampering their location in physician shortage areas where they were particularly needed, and increasing cost of care [13–15]. Now that substantial evidence points to the safety of NP care [6], later adopters of NP roles can benefit from early policy lessons to avoid unnecessary restrictions in NP scope-of-practice that are politically controversial and costly to change.

### Implications and lessons from the United States

The U.S. has the longest experience with NPs, dating back more than 50 years. In the U.S., the 50 states have jurisdictional authority over licensing, scope-of-practice laws and other regulations that directly impact on health professionals. State-specific variations in NP regulatory and financing policies provide a rich experience base from which to draw policy lessons that could benefit countries like Israel now considering adoption of expanded roles for nurses. A shortage of primary care was the initial impetus for the evolvement of NP roles in the U.S. Yet, over time the adaptability of NPs has contributed to their increased use in a variety of innovative roles in healthcare, suggesting that the NP role will continue to evolve to adapt healthcare to changing patient needs. Three policy lessons emerge.

First, after decades of research on the effectiveness and quality of NPs, the focus of research and policy debate in the U.S. is shifting away from whether NP-provided care is safe, to how to reduce barriers to practice and maximize access for those most in need, such as Medicaid beneficiaries and vulnerable groups. Since health system design is shifting in fundamental ways in the U.S., NPs are increasingly required as part of teams to address unmet need as well as innovations to improve transitions from hospitals to home and the reduction of unnecessary readmissions especially for elderly and chronically ill [6].

Second, numerous studies have analyzed the effects of regulation and scope-of-practice laws on NP distribution, access for patients and healthcare costs [14], suggesting that restrictive regulations have potentially large unintended consequences. In an analysis of more than 250,000 ambulatory medical practices, NPs in U.S. states allowing full scope-of-practice for NPs had a 13 % increased odds of working in primary care [15]. In states allowing for full NP scope-of-practice and reimbursement at the same rates as physicians for the same primary care services, NPs were 20 % more likely to practice in primary care. In states with NP supportive regulation and payment policies, access to primary care was significantly improved for Medicaid beneficiaries who often experience long waiting times for a primary care appointment [15]. Additionally, cost of office visits were lower in states with full NP practice authority and full payment for NP services [13].

Third, the evolvement of NP roles with high levels of clinical decision-making and salaries, seems to have contributed to increased attractiveness of nursing as a career [16]. Nursing in the U.S. is one of the most popular career choices, in direct contrast to trends in other countries, including Israel, where interest in nursing is much lower. Graduations from U.S. nursing schools have doubled from 75,000 to 150,000 a year in the past 15 years [16], and thousands of qualified applicants to nursing schools are being turned away. As pointed out by Auerbach et al. [16], several factors may have contributed to this remarkable growth, including a national media campaign to promote nursing, increased growth in overall healthcare spending, and dynamic growth in the number and type of nursing education programs. There has been a substantial increase in the number of second degree students entering nursing, with BAs and higher degrees in other fields.

Countries newly implementing NP roles are concerned that the availability of nurses that are already in short supply will be further eroded by task-shifting from doctors to nurses. The U.S. experience suggests the opposite. Creating more jobs for NPs, opportunities to lead innovative care programs in teams, and better access to higher nursing education, may stimulate greater interest in nursing and serve as a catalyst for increasing the overall supply of nurses.

## Conclusions

Many countries are considering introducing expanded roles for nurses that include the transfer of responsibilities from physicians to nurses with advanced education and clinical expertise. The evidence on their effectiveness and safety is compelling. There is much to be learned from ‘early adopter’ countries, such as the U.S., to avoid some of the unintended consequences of policies and politics that hampered implementation and uptake.

Policy lessons with cross-country relevance are three-fold. First, governance and regulation are critical policy levers: without official authorization of expanded scope-of-practice, nurses cannot practice in advanced roles officially and legally. Second, payment policies are important as they determine if services will be reimbursed and at what level. Third, expanding the roles of nurses may contribute to an enhanced public view of nursing as a career option.

In Israel and other countries in early stages of reforms, many including Aaron & Andrew, suggest that team-based care in which physicians are presumably in charge is an organizational strategy to manage variations in education and competencies among the emerging non-physician providers. Research from early adopters, however, points to the pitfalls of trying to codify a professional responsibility through regulation, such as requirements that NPs have formal collaborative agreements with physicians. There is now sufficient evidence that requiring physician supervision has no effect on quality of care but does increase the cost of care and adversely affects access especially for vulnerable populations and rural residents [15]. Advanced practice nurses can potentially make a contribution in any clinical setting, but their impact on improving access to care will be limited if national policies make it difficult for these new providers to work outside of hospitals. Aaron & Andrew note that so far in Israel educational initiatives and policies are favoring the employment of advanced practice nurses in hospitals where their potential contribution to improving care may not be optimized. Expanding nurses' roles has the potential to improve access to and quality of care, yet, requires a policy environment that enables, rather than hinders, new role uptake in response to patient needs.

## Abbreviations

BAs, Bachelor of Arts; NPs, Nurse Practitioners; U.K., United Kingdom; U.S., United States.
